# An astrobiological perspective on microbial biofilms: their importance for habitability and production of detectable and lasting biosignatures

**DOI:** 10.1128/aem.01778-24

**Published:** 2025-02-10

**Authors:** Sarah Gonzalez-Henao, Matthew O. Schrenk

**Affiliations:** 1 Department of Microbiology, Genetics, and Immunology, Michigan State University 3078 , East Lansing, Michigan, USA; 2 Department of Earth and Environmental Sciences, Michigan State University 3078 , East Lansing, Michigan, USA; University of Wisconsin-Madison, Madison, Wisconsin, USA

**Keywords:** biofilms, astrobiology, habitability, biosignatures, extreme environments, planetary analogs

## Abstract

The search for life elsewhere in the universe has remained one of the main goals of astrobiological exploration. In this quest, extreme environments on Earth have served as analogs to study the potential habitability of Mars and icy moons, which include but are not limited to hydrothermal vent systems, acid lakes, deserts, and polar ice, among others. Within the various forms that life manifests, biofilms constitute one of the most widespread phenotypes and are ubiquitous in extreme environments. Biofilms are structured communities of microorganisms enclosed in a matrix of extracellular polymeric substances (EPS) that protect against unfavorable and dynamic conditions. These concentrated structures and their associated chemistry may serve as unique and persistent signatures of life processes that may aid in their detection. Here we propose biofilms as a model system to understand the habitability of extraterrestrial systems and as sources of recognizable and persistent biosignatures for life detection. By testing these ideas in extreme analog environments on Earth, this approach could be used to guide and focus future exploration of samples encompassing the geologic record of early Earth as well as other planets and moons of our solar system.

## INTRODUCTION

**Fig 1:**
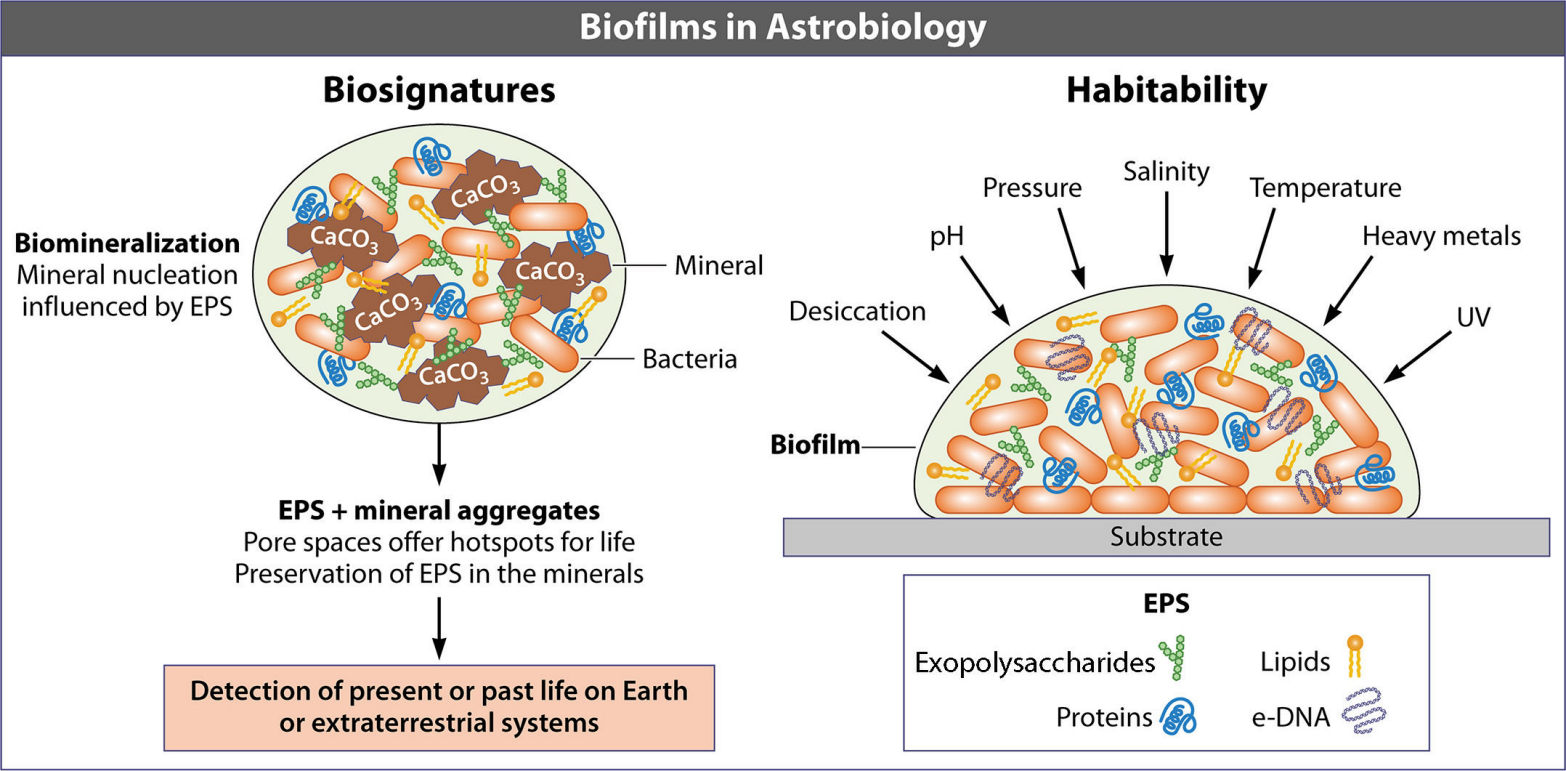
Schematic diagram of the implications of microbial biofilms for astrobiology. Biofilms expand the habitability of microorganisms by shielding them from extreme conditions. This protective function is largely attributed to the EPS, which comprises exopolysaccharides, proteins, lipids, and e-DNA. In addition, biofilms are a promising source of biosignatures, as some components of EPS are highly resistant to degradation. EPS play a crucial role in biomineralization, the process by which microorganisms facilitate mineral formation, often in discrete and easily discriminated structures. The interactions between EPS and minerals further contribute to the preservation of these biopolymers, making them valuable indicators of microbial life on Earth and in extraterrestrial environments.

Microorganisms withstand a wide range of environmental stresses which include high-intensity ultraviolet (UV) radiation, high or low temperature, high alkalinity and acidity, high salinity, high and low pressure, poor nutrients, toxic metals, antibiotic compounds, and desiccation, among other factors (Fig. 1) (1). Microorganisms surviving under these extreme conditions exhibit resistance mechanisms, and in most of these cases, the role of the biofilm phenotype is considered to be a crucial component of their success (1). Biofilms are one of the most widely distributed and successful phenotypes of life on Earth and consist of aggregations of microbial cells associated with abiotic or biotic interfaces that are enclosed in a matrix of extracellular polymeric substances (EPS) that provides structural support (2–4). EPS are organic polymers of biological origin that moderate the interaction of microbial cells with their environment and are comprised of polysaccharides, proteins, extracellular DNA (eDNA), and lipids (5). The organization and structure of the biofilm are based on interactions between cells and the EPS (3). These interactions also determine the mechanical properties of the matrix and the physiological activity of microorganisms within the biofilm (3). In addition, EPS forms a three-dimensional scaffold that confers protection to the cells, facilitates cell-cell communication, and provides structural stability to the biofilm (6).

Biofilms comprise a wide range of structures that are influenced by diverse biological factors such as microbial community composition and population growth rates, the production and composition of EPS, and also by the surrounding environment. For instance, biofilms form columnar towers and mushroom-shaped microcolonies under low laminar flow conditions, or filamentous streamers under high flow conditions that are common in hot springs or acid mine drainage runoff (2). If the environmental conditions are favorable, bacteria can attach permanently to surfaces and secrete EPS, establishing a strong bond with the surface (irreversible adhesion), where the attached bacteria continue to secrete EPS and create a micro-niche that enhances their survival and proliferation, allowing colonization by other bacteria (7). As microbial diversity in the biofilm increases, competition for resources becomes predominant and the communities tend to change over time (succession), which may be facilitated by niche diversification and recycling of resources (8). Interestingly, adhesion to surfaces, such as minerals, promotes bacterial growth, particularly in nutrient-poor environments (9). It provides a site for extracellular nutrient transfer to the biofilm while shielding bacteria from mechanical damage and shear forces caused by fluid flow (9). In addition, bacteria can acquire metabolites and cofactors directly from the surfaces they adhere to, further supporting their growth (9). The interactions of EPS with mineral surfaces result in significantly stronger adhesion of the biofilm and increased biofilm and surface stability, which contributes to the preservation of the biofilms over time (8).

Biofilms are important in understanding microbial survival and growth under extreme conditions (1, 10–13). For instance, an important property of the biofilm conferred by the matrix is tolerance to desiccation. Bacteria in the biofilm adapt to desiccation by generating EPS molecules that form a hydrogel that retains water due to the substantial amount of hydrated polymers within the matrix (3, 14). Biofilms are also reservoirs of phenotypic and genetic diversity that allow microbial communities to adapt to extreme and changing conditions and work collaboratively (15). Most of the microbial communities found in extreme environments are distributed and assembled in extensive biofilms and microbial mats (16). Consequently, they are highly relevant for astrobiology, a discipline dedicated to studying life beyond Earth, understanding the origins of life on our planet, and seeking evidence of life in other parts of the universe (17). Literature on biofilm characterization is extensive; however, a large part of it is focused on antibiotic resistance and biomedical research (18–21), or applications in wastewater treatment (22–25). Therefore, this review aims to offer a novel perspective on biofilms within the field of Astrobiology (Fig. 1), focusing on their implications for creating habitable conditions for microbial life and their potential as biosignatures for life detection. This review will explore the importance of biofilms in relation to habitability, their interaction with the environment, mineralization processes, origins of life, biosignatures, and structural biofilm adaptations in Earth analog environments. In addition, we discuss relevant techniques to study biofilms in Astrobiology missions.

## THE ROLE OF BIOFILMS IN HABITABILITY

As discussed earlier, biofilms are strongly associated with the ability of microorganisms to inhabit the diverse and extreme environments across modern-day Earth. By understanding how biofilms can enable the growth and survival of microbial life on Earth, it is possible to gain insights into the habitability of other planets and moons that could help guide the search for extraterrestrial life, past or present. Habitability is defined as the ability of an environment to support the activity of at least one known organism (26). Experiments with microorganisms under extreme environmental conditions including variations in temperature (27, 28), pH (28), salt concentrations (27, 28), UV irradiation (10), desiccation (29, 30), and high concentrations of metals (31) have shown the formation of biofilms and support the observations that biofilms provide protection and increase survival of microorganisms exposed to unfavorable environmental conditions. Biofilm formation is commonly discussed as one of the main strategies by microorganisms to withstand multiple extremes, including space-like conditions (32). It has been demonstrated that EPS contributes significantly to this resistance as they are critical in establishing the physical and functional properties of a biofilm, and are involved in the ecological adaptation of microbial communities to their host environments (16, 28).

Some of the biofilm adaptations to diverse environmental conditions (Table 1) include the following. In dry environments, it has been shown that microorganisms allocate resources for increased EPS production to alter their microenvironment and enhance survival from desiccation (33). In hypersaline mats, EPS buffer cells against either desiccation or rapid changes in water potential (34). In alkaline environments, the acidic components of EPS form a barrier that reduces proton permeability, helping to shield the biofilm from adverse pH conditions (28). EPS provide a ∼10 µm thick layer around the cells within the center of the flocs, capable of growth at pH values of 11.0 and 11.5, maintaining internal pH values of 10.4 and 10.7 (35). In the case of acidic environments, increased production of EPS has been observed as well as an enhanced ability to bind metals (36). At a moderately low pH (pH 5.0), *Pseudomonas aeruginosa* produces a thicker biofilm with higher biomass (37). Furthermore, it causes a decrease in the inner membrane permeability and increases its viscosity (37). In addition, biofilms utilize acetoin biosynthesis as a form of active pH regulation to maintain pH homeostasis and minimize cellular stress (38). Biofilms can also modulate their extracellular pH to the preferred neutrophile range, even when starting from acidic and alkaline initial conditions, while planktonic cells cannot (38).

**TABLE 1:** Biofilm adaptations to diverse environmental stresses

Environmental stress	Adaptation
Desiccation	Increased EPS production (33) EPS buffer cells against either desiccation (34)
High pH	EPS act as a barrier limiting the proton permeability (28) EPS provide a ∼10 µm thick layer around the cells maintaining internal pH values of 10.4 and 10.7 (35)
Low pH	Increased production of EPS and ability to bind metals (38) Acetoin biosynthesis for active pH regulation (38)
Metals and heavy metals	EPS bind iron to support growth and biofilm formation (39) EPS protein component mediates absorption of manganese (40) Increased EPS production contribute to the sequestration of metals (41–44) EPS serve as a natural organic ligand that could bind dissolved metals (44)
High salinity	Biofouling on membrane surface and overproduction of EPS (12)
High pressure	EPS matrix conveys protection against mechanical challenges (45) Increased EPS production, particularly in polysaccharides (45)
High temperature	Accumulation of thermoprotectants (28) Formation of a thick, stable biofilm in some bacteria (46)
Low temperatures	Formation of stable emulsions (47) EPS serve as cryoprotectants for the cells (48, 49) EPS modify the local environment in sea ice brine channels (48)

Another stressor encountered by microorganisms in extreme environments is variable heavy metal concentrations (Table 1). Some metals involved in biofilm formation include iron and manganese, and when these elements are limited, EPS can bind to the metals and support biofilm formation (50, 51). For instance, exopolysaccharides like Psl can bind and sequester iron to serve as an iron pool or an iron storage channel in a biofilm, stimulating its formation (39). Furthermore, EPS-mediated adsorption was the main mechanism behind manganese accumulation by periphytic biofilms (40). In the presence of heavy metals, biofilm formation and increased EPS production contribute to their sequestration, including arsenic (41), mercury (42), cadmium (43), copper (44), and lead (44). EPS serve as a natural organic ligand that can bind dissolved copper and lead under varying free metal ion concentrations and pH (44). In saline environments, bacteria have a high potential to cause biofouling on the membrane surface as the bacteria still maintain the cell activity and overproduce EPS (12). In the case of pressure, the viscoelasticity of a biofilm’s EPS matrix conveys protection against mechanical challenges (45). High pressure leads to increased EPS production, particularly in polysaccharides, where there is higher resistance to exerted mechanical force due to an immediate increase in polysaccharide content (45).

Finally, under high temperatures, the matrix promotes the accumulation of thermoprotectants formed by microorganisms within the biofilm (28). *Bacillus cereus* WPySW2 can form a thick, stable biofilm under high temperatures, which causes an ecological function to change from being a probiotic to accelerating disease in algae (46). Conversely, under low temperatures, EPS from Antarctic bacteria showed an ability to form stable emulsions, protect cells from freeze-thaw cycles, and chelate heavy metals (47). Interestingly, EPS serve as cryoprotectants for the cells, they can modify the local environment in sea ice brine channels and form a chemically diverse source of dissolved organic matter to the water column upon ice melt (48). In Table 1, we summarize the above-mentioned adaptations of biofilms to the diverse environmental stresses.

The significant role of biofilms in habitability under extreme environmental conditions is also demonstrated by the increased survival ability of biofilm cells compared to that of planktonic cells exposed to a set of unfavorable conditions (28, 52–56). Panitz et al. (56) demonstrated that under diverse stress factors, including desiccation, temperature oscillations, vacuum, a Mars-like gas atmosphere, pressure, and UV radiation, the culturability of *Deinococcus geothermalis* decreased, but was better preserved in the biofilm consortium than in planktonic cells (56). Living within biofilms is key to survival, and the EPS play an essential role in this regard. For instance, Billi et al. (55) demonstrated that, unlike biofilms from *Chroococcidiopsis* strains, multilayered planktonic samples lacked abundant EPS, which contributed to the accumulation of damage and led to a reduced endurance under space and Mars-like conditions (55). In another study, confocal laser scanning microscopy (CLSM) imaging of dried biofilms revealed the presence of a well-developed EPS matrix with abundant lipidic compounds, while dried multilayered planktonic cells showed a reduced lipidic content (55). In addition, it was found that *Pseudomonas aeruginosa* cells, encased in an alginate matrix, exhibited enhanced resistance to UV radiation (54). The alginate matrix absorbed UV light, leading to a higher survival rate compared to planktonic cells exposed to the same levels of UV radiation (54).

Besides these resistance mechanisms, biofilms also exhibit resilience toward different stresses which can be attributed to different factors within the microbial biofilm. The first factor is the EPS network as demonstrated by Zhang et al. (57). The authors showed that the EPS network protects the embedded cells from environmental challenges by providing mechanical resilience in response to large mechanical perturbations (57). A second factor is the species diversity within the biofilm. In another study, the authors analyzed the biofilm microbiomes of sand biofilters and how they respond to increased Mn(II) load. Interestingly, high species richness enabled Mn(II) removal, demonstrating the microbiomes’ resilience in the face of short-term increases in Mn(II) load (58). Furthermore, Feng et al. (59) showed that biofilm communities studied in small bioreactors called microbial electrolysis cells (MEC) could recover to stable performance after pH disturbance, exhibiting a great resilience ability (59). In addition, those with higher diversity tended to recover faster, implying biofilms with high biodiversity showed better resilience in response to environmental disturbance (59). A third factor involved in the resilience of biofilms is environmental heterogeneity. Dzubakova et al. (60) demonstrated that in streambed landscapes, roughness, and exposure to water flow promoted biofilm carrying capacity and growth trait diversity, which suggests that the environment selecting for adaptive capacities prior to disturbance (i.e., memory effects) and biofilm connectivity into spatial networks (i.e., mobile links), contribute to the spatial resilience of biofilms in streambed landscapes (60).

A clear example of resilience and resistance is demonstrated by McKew et al. (61). The authors examined microbial biofilms exposed to periods of desiccation and rewetting (intertidal zones) and showed that within the initial 5–10 days of drying, biofilm functionality undergoes significant changes, including cells avoiding the surface, decreased photosynthesis, and reduced metabolic activity, but no major shifts in the microbial community composition (61). Reflooding resulted in a rapid recovery by the benthic diatoms and biofilm activity (a high level of resilience with respect to functioning), but the formation of a different bacterial community after prolonged drying (61). In this regard, a temporal sequence of effects of desiccation and rewetting was observed, and the most notable feature was the overall resistance and resilience of the microbial community (61). The findings discussed demonstrate that microorganisms exposed as biofilms have an increased chance of survival under extreme conditions on space and Mars compared to the planktonic lifestyle, where a microbial community capable of resisting diverse environmental stressors but also resilient to drastic changes, provides strong foundations for exploring the potential for life on Mars and other planets.

## ENVIRONMENTAL INFLUENCES ON BIOFILM STRUCTURE AND COMPOSITION

Environmental conditions play a crucial role in shaping the structure and composition of biofilms (16, 62). Blanco et al. (16) showed that the external environment strongly influences biofilm characteristics, and their results highlight the existence of conserved EPS compositional patterns for each extreme environment (acidic, cold, and thermal habitats) (16). For instance, in acidic environments such as Rio Tinto and other acid mine drainage sites, biofilms showed significantly higher EPS content by dry weight and metal content (16). This metal absorption capacity is largely due to the presence of carbohydrates in the biofilms, its functional groups, such as carboxyl, phosphate, and sulfate moieties, and their ligand-binding preferences for specific metals (16, 36, 63). By contrast, in cold environments, it was observed a significantly higher DNA and sugar contents, with the sugars functioning as cryoprotectants at low temperatures (16, 47), or possibly, acting as ligands for trace metal nutrients as previously described in Table 1 (64). The structure and composition of the biofilm is described as an “amalgam of complexity, heterogeneity and variability” which is relevant in defining the biofilm properties (14). For instance, acetyl groups are common substituents of exopolysaccharides like alginate, enhancing the adhesive and cohesive properties of EPS (65). This modification significantly influences bacterial aggregation into microcolonies and contributes to the heterogeneous architecture of mature biofilms (65). In addition, the interaction between anionic EPS containing carboxylic groups and multivalent cations, such as Ca²^+^, can further shape biofilm architecture. These cations form bridges between polyanionic alginate molecules, promoting the development of thick, compact biofilms with increased mechanical stability (66).

It is worth noting that not only does the environment exert a role in shaping the composition of the biofilms but the biofilm producers can shape the environment for increased habitability. Noffke (67) describes the EPS produced by microbes as “the world they built, the world they live, eat, fight, multiply, and die in” (67). These biopolymers are the main player in shaping the environment as shown in different studies. Krembs et al. (68) examined the effects of algal EPS on the microstructure and salt retention of ice grown and found that the exopolymers of *Melosira arctica* led to more disordered ice crystals, greater pore density, more complex pore geometries, and greater salt retention by the newly formed ice, demonstrating that EPS modifies the ice and pore microstructure, which improves sea ice habitability, survivability, and potential for increased primary productivity (68, 69). Furthermore, De Los Rios et al. (2003) proposed that in Antarctic rocks, biofilm structure could favor the formation of microsites with specific physicochemical conditions appropriate for the survival of microbial communities in this extreme environment, as the Antarctic biofilms studied were characterized by the presence of EPS and acidic microenvironments in the proximity of the cells (70). Other relevant properties of the biofilms that shape the environment include the capacity of EPS to mediate the formation of organo-mineral associations in soils, which affects the composition of immobile and mobile organic matter and the reactivity of minerals (71), their capacity to aggregate mineral particles, enhancing their cohesion and their ability to retain water (69, 72), and their ability to stabilize sediments (biostabilization), which become more resistant to erosion and represent an excellent substratum for biofilm growth (73). Consequently, EPS producers serve as “ecosystem engineers,” in the context of their ability to modulate the availability of resources by causing physical changes in biotic or abiotic materials, modifying, maintaining, or creating habitats (74), as they improve environmental habitability and enhance survival by microorganisms. These properties of EPS are relevant for astrobiology since they play a direct role in the maintenance and resilience of microbial life in extreme environments.

A relevant aspect of biofilms and habitability relates to colonization and succession, considering that the ability to attach to surfaces and form biofilms provides bacteria with important advantages including (i) increased access to nutrients, (ii) protection against unfavorable environmental conditions, and (iii) shelter from predation (75). The attachment of free-swimming cells to a surface is highly regulated by environmental cues (76). For instance, microorganisms tend to form biofilms under oligotrophic or starvation conditions (76, 77). In another study, Zhang et al. (78) demonstrated that EPS production can be triggered at the single-cell level by reducing nutrient concentration but can also increase at a critical colony thickness that depends on the initial amount of carbon sources in the medium (78). In this regard, the colonization of a surface and formation of biofilm could provide information about the habitability of an environment and how biofilms provide an essential protective niche for microorganisms, also referred to as the “protective clothing” in extreme environments (1).

**Fig 2:**
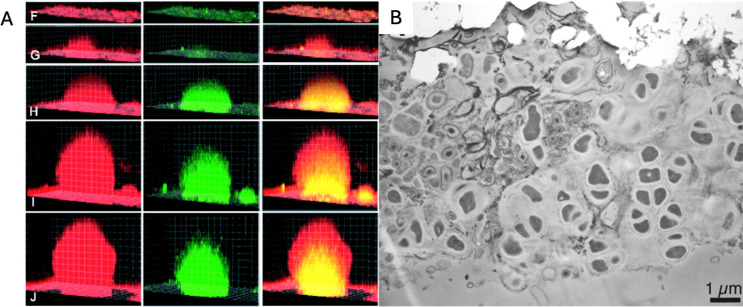
**(A**) Representative developmental course of the *S. oneidensis DKN312* biofilm. In the first column, the cells are constitutively expressing the gene *ecfp—*whose fluorescence is false red. In the second column, the cells expressing the *mtrB* reporter are green, a gene expressed under low-O_2_ conditions. The third column is an overlay of the red and green channels (82). Overall, panels F to J show that *mtrB* was not expressed at the early stages of biofilm development, which is consistent with full O_2_ availability in structures less than 60 µm in diameter. Interestingly, *mtrB* expression appeared in the interior spatial domains of biofilms only at late developmental stages in structures more than 100 µm in diameter, when the interior cells were likely O_2_ limited (82). (**B**) Transmission electron microscopy (TEM) of carbonate chimney thin sections. The prevalence of a cell type with sarcinal morphology is observed but also multiple cell types are present and closely associated with each other. In addition, a viscous matrix appears to surround each cell cluster and may aid in the attachment of cells to the carbonate minerals (bright white areas) (83). Image A was adapted from reference 82. Image B was adapted from reference 83. No changes were made to the original images.

Biofilms are dynamic systems that besides colonizing surfaces, experience succession, a process by which species composition of biological communities changes over time (79). Lee et al. (80) investigated biofilm succession in Antarctic marine environments and observed that the bacterial community composition in the biofilms changed drastically during the early stage of biofilm formation, which supports the views that bacterial community composition of biofilm is niche specific and influenced by interactions with the surrounding environment (80). Interestingly, *Pseudoalteromonas prydzensis* played a significant role in the maturation of biofilm, whose EPS can control bacterial attachment and serve as antibacterial components that enhance the survival of other organisms (80, 81). Moreover, the dynamism of these biofilm communities is clearly reflected in the study by Teal et al. (82). It was commonly thought that subpopulations of cells within biofilms are not metabolically active and are “dead”; nevertheless, in this study, reproducible spatiometabolic stratification in *Shewanella oneidensis* biofilms was observed, which means that cells that are maintained in a nongrowing state, are still capable of synthesizing proteins that can act as a reservoir of survival (Fig. 2A) (82). In this way, it appears to be a major interior domain of biofilms where cells generate energy although they are not growing, and these cells have different metabolic activities as a result of the associated microenvironment within the biofilm (82). In another study, Brazelton et al. (83) discovered that cells within single-species biofilms from Lost City Hydrothermal Field (LCHF) may have differentiated into multiple cell types that have different physiological roles and form multicellular communities, which implies a kind of niche partitioning (Fig. 2B) (83). Particularly, differentiation and syntrophy likely evolved as adaptations to maximize the metabolic potential of Lost City habitats including hydrogen and methane utilization (83).

These studies of biofilm succession in extreme environments highlight the influence of the environment in shaping the biofilm and that community turnover within the biofilm contribute to the improved habitability at the extremes, as new niches are created that promote a quick adaptation of the community to the surrounding environment. Furthermore, biofilms are a metabolically active, dynamic, and successful lifestyle where cells are capable to adopt different physiological roles that allow for the survival and efficiency of the biofilm community. Consequently, biofilms represent an excellent model for studying habitability and the diversification of life to Earth’s environments.

## BIOFILMS AS MINERAL SCULPTORS

The association of biofilms with interfaces is relevant for astrobiology as bacterial cells tend to adhere to surfaces and gradually form biofilms, thus facilitating mineral weathering and favoring bacterial proliferation (84). Microbes are not only capable of influencing reactions leading to the dissolution of minerals, but also the formation of new minerals, a process known as biological mineralization, or biomineralization (85, 86). One notable example of this process is microbially induced carbonate precipitation (MICP) (87). In this case, microbial EPS can trap and bind remarkable amounts of calcium to accelerate calcium carbonate precipitation by acting as a physical substrate for mineral nucleation, which is facilitated by the enzymatic hydrolysis of urea in some bacteria like *Bacillus sp* (88). This reaction produces carbonate and ammonia, increasing the pH and carbonate concentration, which then combines with environmental calcium to precipitate as calcium carbonate (88).

**Fig 3:**
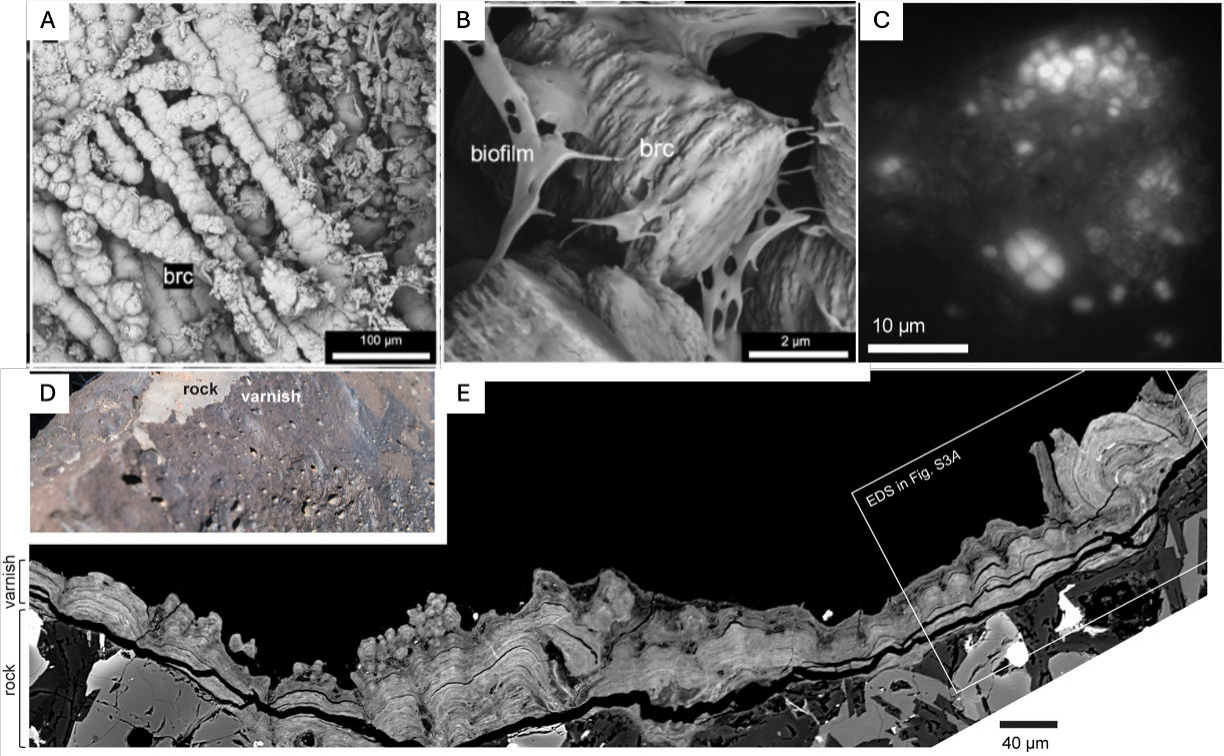
Biomineralization cases of study. (A) Chains of brucite spheres likely formed from the mineralization of microbial filaments (90). (**B**) Stack of brucite plates associated with a partially preserved matrix of EPS (90). (**C**) Fluorescence microscopy highlighting cells of *Chroococcidiopsis* in varnish (92). (**D**) Photograph of varnished surface and underlying rock (92). (**E**) Scanning electron microscope (SEM) image of a cross-section through the varnish–rock interface showing laminations that establish stromatolitic columns and domes (92). Images adapted from references 90 and 92 under a Creative Commons Attribution License (CC BY). No changes were made to the original images.

In the serpentinite-hosted LCHF and Prony Bay Hydrothermal Field (PHF), analogs of seafloor environments on the Icy moons Enceladus and Europa (89), there is evidence for biologically influenced mineralization. In the LCHF, brucite was observed to precipitate directly on microbial filaments, on EPS, as well as on the surfaces of organisms, which suggested that the orientation of the crystal growth of brucite crystals is influenced by the biofilm (Fig. 3A and B) (90). In PHF, Pisapia et al. (91) discovered that bacteria belonging to *Firmicutes,* along with bacteria from the phyla *Acetothermia* and *Omnitrophica,* are the first chimney inhabitants, and are involved in the construction and consolidation of carbonate structures through organomineralization processes, as they are predominant in most juvenile and nascent hydrothermal chimneys and are considered representatives of endolithic serpentinization-based ecosystems (91).

Interestingly, biomineralization can be either biologically controlled or biologically induced/influenced. This process can be seen as an evolutionary advantage, as it allows organisms to enhance their efficiency in utilizing environmental resources for energy, thereby supporting population growth (93). These processes of EPS-mediated biomineralization are significant for astrobiology, as they can create microenvironments that protect microorganisms from harsh conditions and promote biofilm colonization, which has important implications for their habitability in extreme environments and extraterrestrial systems. Furthermore, the chemistry and structure of biomineralization products could also serve as biosignatures for detecting life. For instance, it has been shown that bacterial EPS changes mineral microstructure and texture in a species-specific manner, which might be used as an identification tool for bacterial calcification in present and past environments (93).

Another interesting case of study is desert varnish, which is a dark rock coating that forms in arid environments and is enriched in manganese, which provides a habitat for microbial life shielded by oxide minerals that absorb UV radiation (94). Lingappa et al. (92) proposed that the activity of extremophilic cyanobacteria (*Chroococcidiopsis*) is a key driver of the varnish ecosystems, as they accumulate significant amounts of intracellular manganese and use it as a catalytic antioxidant, an adaptation for coping with the oxidative stress of the arid environment (92). When the bacteria die, their biomass provides a manganese source that is oxidized to form the oxide mineral that contains the varnish (Fig. 3C through E) (92). In addition, since it forms over timescales of millennia, varnish could be considered as a mineral biosignature, and could provide evidence of past microbial life on Mars, as there is evidence for desert varnish formation in this planet from Viking and Pathfinder landing sites (95, 96).

A robust example of biomineralization is the microbialites (benthic microbial carbonate deposits) discovered in a hypersaline alkaline lake on Eleuthera Island (Bahamas) (97). These deposits highlight the critical role of EPS in biomineralization, demonstrating how it can both facilitate and inhibit carbonate formation. In the study, the authors observed that acidic macromolecules in the EPS can inhibit carbonate precipitation leading to non-calcifying mats in the lake (97). This inhibition occurs when the EPS matrix acts as a “cation sponge,” interfering with carbonate formation by removing Ca^2+^ from the solution (97). On the other hand, they observed that precipitation is predominant in shallower depths, and is achieved when calcium availability exceeds the binding capacity of the EPS and/or the binding capacity is reduced through decarboxylation (97). Degradation of EPS can occur by fermentation or UV light, which leads to hydrolysis or the decarboxylation of EPS, and the formation of low molecular weight compounds that support the growth of bacteria (97). This process liberates cations and results in an internal increase in the concentration and availability of calcium and magnesium, leading to the calcification of EPS (97).

## BIOFILMS AS BIOSIGNATURES FOR LIFE DETECTION

Although Earth has undergone drastic environmental changes over the past 4.5 billion years, not all planets and moons may have undergone a similar trajectory or evolved to a similar extent. Therefore, it is important to consider the type of biofilms present at different stages of planetary evolution. Biofilms may have provided homeostasis under fluctuating and unfavorable conditions on the primitive early Earth exposed to extreme temperatures and exposure to UV light and facilitated the origins and early evolution of life (98). Different hypotheses have been proposed on the origins of biofilm formation and their implications for the origin of life on Earth. A common aspect is the intimate association of biofilms with mineral surfaces, in which chemical reactions take place and provide electron acceptors, electron donors, and energy (99–101). In addition, physical proximity or protocells and primitive metabolic network are also important themes to these emerging models (102–107).

In the origins of life, minerals might have played an important role, as they have a wide range of properties that might have contributed to the synthesis and self-assembly of the protocells, polymerized monomers into polymers of the biomolecules, and catalyzed transmembrane redox reactions (107). As stated by Hazen et al. (108) mineral surfaces may have concentrated and helped to organize biomolecules on the early Earth, promoting the transition from a dilute prebiotic “soup” to highly ordered local domains (108). Interestingly, various common rock-forming minerals have been studied as possible templates for organic adsorption. For instance, calcite and quartz represent plausible templates for prebiotic selection and organization of polypeptides (109). Furthermore, the Last Universal Common Ancestor (LUCA) is conceived not as single-celled organisms but rather a community, which leads to the biofilm mode of life and is thought to have emerged from inorganic templates with the active contribution of inorganic surfaces to the development of metabolism and as a cell template (102).

Mineral–microbe interactions leave characteristic and emblematic signatures in rock records, including their morphology, mineral composition and structure, elemental and isotopic fractionation, and recalcitrant organic compounds (110). Some of the components of EPS are highly labile carbon forms, while others, appear quite refractory to degradation, such as certain proteins, peptides, and lipids (34, 111). Some of these refractory components include amyloid fibrils, defined as any fibrillary polypeptide aggregate having a cross-β-quaternary structure (112)

which may be formed from many different proteins and peptides and are a generic structure of the peptide chain (34). For instance, Romero et al. (113) found that the major protein component of the *Bacillus subtilis* biofilm matrix, TasA, forms amyloid fibers, which could play an important function in supporting the structure of the biofilm (113). These refractory components of EPS and distinctive interactions with minerals may serve as potential biosignatures for identifying extraterrestrial life. Biosignatures refer to any object, substance, and/or pattern whose origin specifically requires a biological agent (114). EPS have been considered potential biosignatures of microbial past life (115–119) and as a biosignature in the form of mineral deposits associated with iron-oxidizing bacteria, where the biogenicity may be indicated by filmy mineralized sheets or accumulation of iron on EPS, leading to the formation of assemblages (120). The preservation of these biosignatures is critical for their detection and interpretation in Astrobiology. Notably, the preservation of EPS can be enhanced by various mechanisms: (i) recalcitrance of specific EPS against enzymatic microbial degradation, (ii) chemical stabilization by interactions between minerals and reactive groups in EPS, (iii) physical protection through the formation of aggregates with minerals, or (iv) reduced microbial enzymatic activity (121, 122). These preservation mechanisms are illustrated by ancient microbial records, such as stromatolites, which are believed to be fossilized remnants of cyanobacterial biofilms (123), the biofilm microcolonies identified in the 3.3–3.5-billion-year-old South African Kornberg formation (124), and filamentous biofilms identified in the 3,235-million-year-old deep-sea volcanogenic massive sulfide deposit from the Pilbara Craton of Australia (125).

Interestingly, the interactions between biofilms and minerals are evident not only in surface environments but also in the subsurface. Duteil et al. (122) presented the first evidence of the preservation of EPS and EPS-mineral aggregates in a 6-m-long sedimentary core obtained from an estuarine point bar in the Gironde Estuary (34). High protein concentrations were detected in some deep horizons from the studied core (e.g., 0.93 m, 4.33 m, 4.76 m) which could be explained by some extracellular proteins produced by bacteria or diatoms, such as amyloid fibrils, which are also highly resistant to heterotrophic degradation (34). In another study, Osburn et al. (126) analyzed the mineral selectivity by biofilms in a deep continental subsurface setting, the Deep Mine Microbial Observatory (DeMMO), suggesting that iron and sulfur-rich minerals drive biofilm colonization at DeMMO (126). Each mineral supports a unique microbial consortium with specific metabolic functions that enable the microbes to exploit or protect themselves from the mineral (127). In addition, Sulfate-Methane Transition Zones (SMTZ) from fractures at 0.5 and 19 meters below the seafloor (mbsf) contained macroscopic pink to orange biofilms associated with the anaerobic oxidation of methane (AOM) (128). Notably, the abundance of biofilms at these depths can be explained by the large pore space available for microbial colonization as it has been shown that the sediment grain size can be a controlling factor of cell densities (128). Similarly, Templeton et al. (129) reported high microbial cell abundances in subsurface serpentinites undergoing active serpentinization from The Oman Drilling Project in the Samail Ophiolite that vary at least six orders of magnitude, from ≤3.5 × 10^1^ to 2.9 × 10^7^ cells/g (129). Some of the highest cell abundances in the cores may be localized along the numerous fractures present, which could host dense biofilms (129). These findings are significant, as they could provide crucial insights for guiding astrobiology missions in identifying potential locations and conditions where signs of life might be detected. In this regard, it is important to study the factors that drive mineral selectivity by biofilms and their interactions under various conditions.

## ADAPTATIVE BIOFILM STRUCTURES ENABLE GROWTH AND PRESERVATION IN VARIOUS PLANETARY ENVIRONMENTS

As noted above, biofilms can take on different structures and compositions in response to environmental conditions, which allows them to adapt to the surrounding environment. This raises an intriguing question: what can we infer about the potential occurrence of biofilms on other planets and moons by studying analogous modern-day Earth environments? Current studies on Early Earth, Mars, and Icy Moons analog environments have shown distinct biofilm structures and compositions as described below:

### Early earth environments

- **The Dresser Formation** is located in the East Pilbara granite-greenstone terrane in Western Australia, which contains some of Earth’s oldest and best-preserved volcanic and sedimentary rocks (130). Noffke et al. (131) described microbially induced sedimentary structures (MISS) from this environment, which result from microbe-sediment interactions and record diverse communities of microbial mats (131). Notably, associations of macroscopic and microscopic MISS can be found in the Dresser Formation which extends the geological record of MISS by almost 300 million years (131). Macroscopic MISS include polygonal oscillation cracks and gas domes, erosional remnants, and pockets and mat chips, whereas microscopic MISS are described as tufts, sinusoidal structures, and laminae fabrics (131). MISS serve as possible templates for the decoding of ancient life processes on Mars, as they represent a window into past life and have been proposed as biosignatures that could be present on this planet, considering that sedimentary rocks form a significant volume of deposits on Mars (132).
- **The Barberton Greenstone Belt (BGB)** in the Kaapvaal Craton of South Africa is an ancient environment that provides a way to study sedimentary depositional environments in the early Earth (133). In the BGB, diverse biosignatures have been described that range from carbonaceous cherts containing microstructures, traces of hydrothermal biofilms, photosynthetic microbial mats, remnants of stromatolites, and microfossils (134). Interestingly, the poor preservation of these structures makes it challenging to determine their biogenicity; nevertheless, they are interpreted and recognized as microbial biofilms and MISS considering that (i) they are fine, crinkly, micro-tufted, laminated structures, (ii) they have micron-scale morphological characters typical of microbial mats, and (iii) they present in-sediment cohesiveness (133). Therefore, the Barberton Greenstone Belt preserves one of the most ancient records of microbial life on Earth.

### Mars analog environments

- **Rio Tinto** (Spain), an extremely acidic environment, has been proposed as a Mars terrestrial analog due to the presence of sulfates and iron oxides, which are products of the bioleaching of iron-containing sulfides and are present in vast amounts on Mars (135). Biofilms at Rio Tinto are unique, compact, and have a well-defined layered structure, including layers of cells loosely packed between layers of minerals (135). The biofilm structure in this environment is attributed to the seasonal changes (rainy season), differences in water velocity, and the amount of material accumulated on the sediments (135, 136). Consequently, microbial biofilms at Rio Tinto are tridimensional structures associated with surfaces that show a spectrum of heterogeneous forms determined by the microorganisms and the environmental conditions (135).
- **The Atacama Desert** is another widely studied Mars terrestrial analog environment, the driest and oldest desert on Earth, which has been a Mars analog model for almost 20 years due to its extreme dryness, the highest UV radiation levels on Earth, and highly saline and oxidizing soils (137). Maria et al (2021) characterized 20 previously unexplored Andean microbial mats and microbialites ecosystems in eight different lakes and wetlands from The Salar de Atacama in the Chilean Central Andes (138). The studied mats and microbialites primarily consist of calcium carbonate minerals, such as aragonite and calcite, along with halite (138). By contrast, the endoevaporites are mainly composed of gypsum and halite (138). Bacteroidetes and Proteobacteria are the dominant phyla found in the carbonate-rich mats and microbialites (138). Furthermore, biofilms studied in this environment have been found in the form of epilithic biofilms covering the rocks in the caves of the coastal range of the desert, which are also analogs of Martian caves. In this cave, two modes of cell aggregations in the biofilm were observed: one in which the cells appear to be loosely associated with each other, with low or no presence of EPS. In this case, the downregulation of EPS production at high cell densities could allow cells to redirect energy from EPS production into growth and cell division prior to a dispersal event (139). In the other form of aggregation, the cells are embedded in a well-developed extracellular matrix, which could facilitate the retention for longer periods of the scarce water available in the cave (139).
- **Antarctica** is characterized by a cold and dry climate, low water availability, salt concentration, desiccation, and high radiation (140). De Los Rios et al. (141) collected two types of biofilms found in granite rocks in Antarctica. One of them was loosely adhered to the substrate and was observed as a cell layer that was attached to the substrate by a thin film of EPS. The other biofilm was tightly attached to the substrate, showed a closer association with the rock minerals, and appeared as a dense matrix in which cells, EPS, and mineral fragments were intermixed (141). Considering these two types of biofilms, the amounts of EPS and their organization may determine the differences in adhesion and effects on the lithic substrate observed in the biofilms (141).

### Icy moons analogs

**Fig 4:**
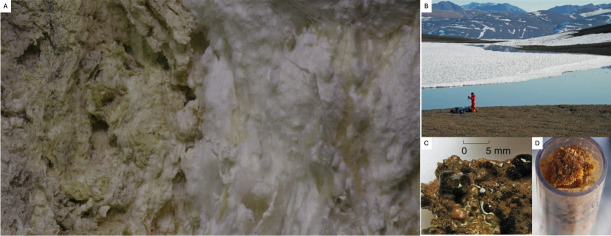
Biofilm structures in analog extreme environments on modern Earth. (**A**) The LCHF: Exteriors of the chimneys coated with biofilms that can be observed along the edges and in the cracks of the chimneys (142). (**B to D**) Perennially ice-covered High Arctic lake: (B) Ward Hunt Lake showing the littoral open water zone (moat) and multi-year ice over the deeper waters of the lake (145). (**C**) Shallow biofilm over the rocks in the moat zone (145). (**D**) Sample of the deep biofilm in mini-Glew sediment core (38 mm diameter) from 10 m depth (145). Image in panel A is courtesy of Susan Lang, University of South Carolina/NSF/ROV Jason/2018 © Woods Hole Oceanographic Institution. Images in panels B to D were adapted from reference 145 under a Creative Commons Attribution License (CC BY). No changes were made to the original images.

- The **Lost City Hydrothermal Vents,** a submarine hydrothermal system, serves as an analog of the ocean worlds of the outer solar system. Microbial life in LCHF is prevalent throughout the Lost City chimneys and is powered by the hydrogen and organic molecules produced by serpentinization, which is also thought to occur on Europa and Enceladus (142). In the Lost City, thick mucilaginous microbial biofilm communities live in the chimneys (Fig. 4A), which are dominated by organisms likely to consume H_2_, CH_4_, and formate (142). Tens of micrometer-thick microbial biofilms composed of irregular coccoid cells, ≈1–3 µm in diameter have been found inhabiting the pore spaces within the carbonate chimneys, where the cells are bound to mineral surfaces in a web-like matrix of microbial exopolysaccharides (143).
- Both the **Arctic and Antarctic** offer locations that mimic environments present on the icy moons of Jupiter and Saturn (144). In these extreme environments, the microbial communities are viable despite the harsh conditions in the terrestrial glaciers and cryo-permafrost, which increases the plausibility of finding life forms in Europa and Enceladus (144). Mohit et al. (145) reported the discovery of biofilms at the deepest site of a perennially ice-covered High Arctic lake, which represents a model of polar microbial communities that remain unfrozen throughout the year (145). These biofilms were collected in the Ward Hunt Lake (WHL), located in the northernmost region of High Arctic Canada, which is fully ice-covered for 10 months of the year, and mid-summer ice thickness can be up to 4 meters (Fig. 4B) (145). The biofilms are described as a microbial community that formed a continuous biofilm over the lake floor at the deep-water location that was a 3–5 mm orange-brown, loosely cohesive layer overlying a 10–15 mm thick beige-colored zone of sediment (Fig. 4D) (145). Furthermore, benthic biofilms can be found in the shallow moat of open water that forms in summer (Fig. 4C) (145).

As previously noted, biofilms from different extreme environments display diverse structures and conformations that are linked to the conditions of the environment they live in, which are potential adaptations that allow them to colonize and thrive effectively in their environment. In these distinct scenarios, biofilms are not defined by one common structure, but rather, they adjust and are dynamic systems that meet energetic demands according to the surrounding conditions. Biofilms can range from tens of micrometers thick as in the hydrothermal vent systems to a single film layer on a rock as in Antarctica. Thus, as we look for life on other planets, it is relevant to keep an open mind on what biofilms might look like, and by studying these life forms from Earth analog environments, it is possible to gain insight into the unlimited diversity of structures and morphologies that could be found elsewhere.

## TECHNIQUES USED TO STUDY BIOFILMS AND THEIR RELATIONSHIP TO ASTROBIOLOGY MISSIONS

Throughout this review, we have described the implications of biofilms in an astrobiology context, ranging from the outstanding properties of biofilms that allow microorganisms to survive in the extremes, to the description of the potential use of biofilms as biosignatures for life detection in extraterrestrial settings. In this quest, the techniques and approaches used to study biofilms on Earth gain particular relevance as they provide a comprehensive understanding and characterization of the structure and composition of biofilms, where commonly, various approaches are used to study biofilms (146). The most relevant include microscopy and spectroscopy approaches as described below:

### Microscopy

#### Scanning electron microscopy (SEM)

**Fig 5:**
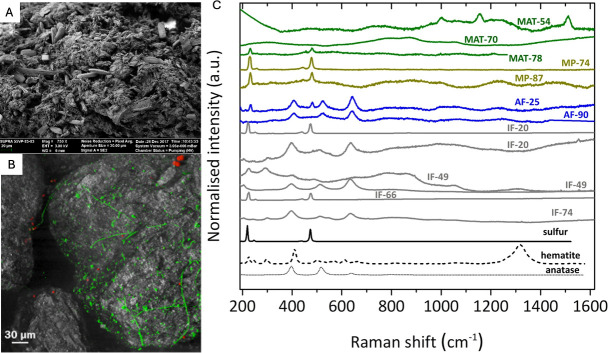
Microscopy and spectroscopy techniques to study biofilms. (**A**) SEM images of endolithic microbial communities (138). (**B**) CLSM images of cryoconite sediment with associated microbial communities and biofilms. In red, the auto-fluorescence cells are observed, green corresponds to SYBR Green stained microbes, and gray represents the reflection of the sediment (149). (**C**) Raman spectra of the minerals identified in the Icelandic hydrothermal regimes; hot spring biofilms (MATs; green), mud pots (MPs; brown), active fumaroles (AFs; blue), and inactive fumaroles (IFs; gray) (150). At the bottom, Raman spectra of minerals are shown in black for comparison and initial mineral identification. Furthermore, the spectrum of MAT-54 corresponds to carotenoids, and the different Raman spectra profiles depending on the spot measured in IF-20 and IF-49 are caused by mineral heterogeneities (150). Images adapted from references 138, 149, and 150 under a Creative Commons Attribution License (CC BY). No changes were made to the original images.

SEM is used for structural analysis through high-resolution imaging, which allows for the evaluation of bacterial interaction, EPS organization, complexation with minerals, and biofilm morphology (135, 141, 147). Considering that the next space missions will investigate the possibility of extinct or extant life on Mars, NASA is developing a Miniaturized Variable Pressure Scanning Electron Microscope (MVP-SEM) for *In-Situ* Mars Surface Sample Analysis, which would answer questions about the petrology, evolution, and habitability of Mars while providing an understanding of the surface environment that will be critical to the success and health of future human exploration (148). In this regard, the MVP-SEM is a valuable tool for the search of the past and present life on Mars and the potential detection of biosignatures produced by biofilms. Furthermore, among the studies using SEM to study biofilms in Earth analog environments include the Atacama Desert. As mentioned before, Maria et al. (2021) characterized Andean microbial mats and microbialites ecosystems from The Salar de Atacama in the Chilean Central Andes and used SEM to study the association of the prokaryotic and eukaryotic microorganisms with the minerals, which revealed the presence of diatoms, filamentous cyanobacteria, and other prokaryotic cells (bacilli) (Fig. 5A) (138).

#### Confocal scanning electron microscopy (CLSM)

CLSM produces high-resolution images of the biofilm architecture in three dimensions and offers versatility by the possibility of adding different fluorescent stains to the sample, for example, it is possible to obtain information on the presence of e-DNA, exopolysaccharides and biofilm viability with high sensitivity, specificity, and resolution (151). Interestingly, the bacterial component of a Space Biofilms project performed on the International Space Station (ISS) consisted of characterizing the morphology and gene expression of bacterial biofilms of *Pseudomonas aeruginosa* formed in microgravity, and one of the tools used for this purpose was CLSM, which allowed to quantify biofilm mass, thickness, and surface area coverage (152). In addition, there have been studies using CLSM in Earth analog Environments such as the Antarctic. Smith et al. (2016) showed via CLSM that microbial communities on glacial surfaces in Antarctica persist as biofilms, showing that their spatial organization promotes efficient transfer and cycling of nutrients but also that biofilm formation leads to the accumulation of organic matter on cryoconite minerals (Fig. 5B) (149).

### Spectroscopy

#### Raman spectroscopy

Raman spectroscopy is a suitable technique that allows for the characterization and identification of biofilm-forming bacterial strains and biofilm matrix composition (153). It represents a nondestructive analytical technique that provides fingerprint spectra and has been used as a reliable methodology for the identification of minerals and organics such as EPS in mineral samples (154). Current missions, such as NASA’s Perseverance rover, make use of a RAMAN instrument called Scanning Habitable Environments with Raman and Luminescence for Organics and Chemicals (SHERLOC), which has been investigating organic compounds in the Martian regolith at the surface and near subsurface in the Mars Jezero crater (155). Interestingly, various studies have used Raman in Earth analog environments to study biofilms. Among them, Sanchez-Garcia et al. (150) explored microbial fingerprints and their associated mineralogy in Icelandic hydrothermal systems analog to Mars to identify potentially habitable locations on that planet, which included four hydrothermal substrates: hot springs biofilms, mud pots, and steaming and inactive fumaroles (150). In this study, Raman spectral differences between samples suggested the possible coexistence of abiotic and biomediated sources of minerals in the Icelandic hydrothermal systems (Fig. 5C) (150). In addition, Raman has been widely used to study past life on Mars by studying fossils. For instance, Osterhout et al. (156) used this technique to determine the deep-UV Raman spectra characteristic of individual microfossils from 14 Precambrian cherts (156). The authors showed that the microfossils with an associated D-band (∼1,350 cm^−1^) had lower thermal maturity and thus, had less altered biosignature, indicative of relatively well-preserved organic matter (156). In this regard, Raman spectroscopy is a promising technique for detecting biogenic materials, including EPS, which may indicate potential habitability and suggest conditions suitable for past or present life.

#### Fourier transform infrared spectroscopy (FTIR)

FTIR is a suitable tool that provides information about inorganic volatile contents and organic constitution (157). FTIR and Raman are complementary techniques commonly used to study biofilms (157). Remarkably, pyrolysis–Fourier infrared spectroscopy (pyrolysis-FTIR) is an instrument that has gained particular attention as it has the potential to be an information-dense method for Mars sample triage, and can produce true positives for organic compounds when they are present in quantities of tens of parts per million (158). This technique involves heating solid samples rapidly (up to 20,000°C s^−1^) to liberate gaseous products that are subsequently characterized and quantified with IR transmission spectra (158). Some studies using FTIR in Earth analog environments include Giant pool fingers from Hidden Cave, New Mexico which contain morphological and geochemical evidence of past biofilm microbial communities (159). In this case FTIR was used to identify fatty acids, proteins, PO_2_-carrying compounds, and polysaccharides spatially related to morphological fossil filaments (159).

As previously discussed, these techniques have been extensively employed in biofilm studies. However, further research is needed on EPS-mineral interactions in extreme environments relevant to Early Earth, Mars, or the icy moons. A multimodal approach is essential for detecting these interactions, as they provide critical insights into past or present life and offer clues about the habitability of these environments. Future studies should also address the potential challenges in detecting these biosignatures and focus on developing new and increased sensitive techniques, as the search for life is constrained by the current capabilities of instrumentation and our understanding of Earth’s analog environments.

## CONCLUSIONS AND FUTURE PERSPECTIVES

The search for habitable worlds and life on other planets comprise two of the pillars of astrobiology research. Understanding life on Earth, the only planet we know of on which life arose and evolved, gives us clues in this quest. Biofilms provide a robust model system that facilitates: (i) the search for life on other planets as the EPS are potential biosignature candidates that can be preserved by minerals and (ii) the study of habitability for microbial life forms under extreme conditions, as biofilms have ubiquitously been found in diverse environments from the early history of life on Earth. As noted, biofilms and minerals have an intimate association that is recorded in the fossil record, and the EPS are the link that mediates this interaction, where not just EPS-producing microbes can build their own microenvironments but also the biofilms they produce can modify the surrounding environment to increase its habitability.

Considering this, we argue that understanding biofilm physiology is essential to astrobiological exploration and the search for life in extraterrestrial systems. Some of the gaps in biofilm physiology that need to be addressed include knowledge about molecular mechanisms that regulate the production of EPS under the diverse environmental conditions and metabolic pathways involved that lead to the increased resilience of biofilms which could be studied in the laboratory under controlled conditions in more depth. Furthermore, more studies of the physiological roles of the species living within the microbial biofilms are needed as stratification and cell differentiation of cells have been observed. The triggers for biomineralization processes and mechanisms on how they mediate mineral transformation are still unclear.

As we study biofilms in Earth analog environments relevant to Mars and the Icy moons, we propose biofilms as hotspots for habitability and microbial survival under extreme conditions that could be found in association with minerals in extraterrestrial systems. These are dynamic systems that adapt to the surrounding environment and can form diverse structures and morphologies on mineral phases to create suitable micro-environments. Therefore, as we look for life and explore Mars and the Icy moons of the Solar System it is important to (i) keep an open mind on how we define biofilms considering the diverse biofilm structures, (ii) use multiple approaches and instrumentation to corroborate the biogenicity of biofilms, (iii) classify a list of minerals that biofilms have selectivity for, which could aid to narrow down the exploration regions on Mars, (iv) examine how minerals influence the preservation of biosignatures generated from EPS using modeling techniques, (v) direct efforts toward the exploration of the subsurface of Mars, as it remains largely unexplored and structured biofilm communities in close association with particular minerals could be present, as it has been shown on the subsurface of Earth (126), and (vi) gain a comprehensive understanding on how biofilms influence habitability in extreme environments. The growing sophistication of both microbiological approaches to study biofilms and the technical capabilities of instruments has us poised to make substantial progress in this critical area of astrobiology but requires a holistic, interdisciplinary perspective.

## EDITOR'S NOTE

This article was handled by Betül Kaçar, who acted as a Guest Editor in consultation with the Editor in Chief, Gemma Reguera.

## References

[1] Yin W, Wang Y, Liu L, He J. 2019. Biofilms: the microbial “Protective Clothing” in extreme environments. Int J Mol Sci 20:3423. doi:10.3390/ijms20143423 31336824 PMC6679078

[2] Stoodley P, Sauer K, Davies DG, Costerton JW. 2002. Biofilms as complex differentiated communities. Annu Rev Microbiol 56:187–209. doi:10.1146/annurev.micro.56.012302.160705 12142477

[3] Flemming HC, Wingender J, Szewzyk U, Steinberg P, Rice SA, Kjelleberg S. 2016. Biofilms: an emergent form of bacterial life. Nat Rev Microbiol 14:563–575. doi:10.1038/nrmicro.2016.94 27510863

[4] Donlan RM. 2002. Biofilms: microbial life on surfaces. Emerg Infect Dis 8:881–890. doi:10.3201/eid0809.020063 12194761 PMC2732559

[5] Di Martino P. 2018. Extracellular polymeric substances, a key element in understanding biofilm 791 phenotype. AIMS Microbiol 4:274–288. doi:10.3934/microbiol.2018.2.274 31294215 PMC6604936

[6] Mahto KU, Priyadarshanee M, Samantaray DP, Das S. 2022. Bacterial biofilm and extracellular polymeric substances in the treatment of environmental pollutants: beyond the protective role in survivability. J Clean Prod 379:134759. doi:10.1016/j.jclepro.2022.134759

[7] Giorgi F, Curran JM, Patterson EA. 2022. Real-time monitoring of the dynamics and interactions of bacteria and the early-stage formation of biofilms. Sci Rep 12:18146. doi:10.1038/s41598-022-22669-0 36307497 PMC9616909

[8] Gerbersdorf SU, Westrich B, Paterson DM. 2009. Microbial extracellular polymeric substances (EPS) in fresh water sediments. Microb Ecol 58:334–349. doi:10.1007/s00248-009-9498-8 19242746

[9] Tuson HH, Weibel DB. 2013. Bacteria-surface interactions. Soft Matter 9:4368–4380. doi:10.1039/C3SM27705D 23930134 PMC3733390

[10] Koerdt A, Gödeke J, Berger J, Thormann KM, Albers SV. 2010. Crenarchaeal biofilm formation under extreme conditions. PLoS ONE 5:e14104. doi:10.1371/journal.pone.0014104 21124788 PMC2991349

[11] Hoštacká A, Čižnár I, Štefkovičová M. 2010. Temperature and pH affect the production of bacterial biofilm. Folia Microbiol 55:75–78. doi:10.1007/s12223-010-0012-y 20336508

[12] Kim LH, Chong TH. 2017. Physiological responses of salinity-stressed Vibrio sp. and the effect on the biofilm formation on a nanofiltration membrane. Environ Sci Technol 51:1249–1258. doi:10.1021/acs.est.6b02904 27995790

[13] Stevens AH, Childers D, Fox-Powell M, Nicholson N, Jhoti E, Cockell CS. 2019. Growth, viability, and death of planktonic and biofilm Sphingomonas desiccabilis in simulated Martian Brines. Astrobiology 19:87–98. doi:10.1089/ast.2018.1840 30048150 PMC6338574

[14] Flemming HC, Wingender J. 2010. The biofilm matrix. Nat Rev Microbiol 8:623–633. doi:10.1038/nrmicro2415 20676145

[15] Flores-Vargas G, Bergsveinson J, Lawrence JR, Korber DR. 2021. Environmental biofilms as reservoirs for antimicrobial resistance. Front Microbiol 12. doi:10.3389/fmicb.2021.766242 PMC8713029 34970233

[16] Blanco Y, Rivas LA, González-Toril E, Ruiz-Bermejo M, Moreno-Paz M, Parro V, Palacín A, Aguilera Á, Puente-Sánchez F. 2019. Environmental parameters, and not phylogeny, determine the composition of extracellular polymeric substances in microbial mats from extreme environments. Sci Total Environ 650:384–393. doi:10.1016/j.scitotenv.2018.08.440 30199683

[17] De Mol ML. 2023. Astrobiology in space: a comprehensive look at the solar system. Life (Basel) 13:675. doi:10.3390/life13030675 36983831 PMC10054531

[18] Bowler P, Murphy C, Wolcott R. 2020. Biofilm exacerbates antibiotic resistance: is this a current oversight in antimicrobial stewardship? Antimicrob Resist Infect Control 9:162. doi:10.1186/s13756-020-00830-6 33081846 PMC7576703

[19] Costerton W, Veeh R, Shirtliff M, Pasmore M, Post C, Ehrlich G. 2003. The application of biofilm science to the study and control of chronic bacterial infections. J Clin Invest 112:1466–1477. doi:10.1172/JCI20365 14617746 PMC259139

[20] Abebe GM. 2020. The role of bacterial biofilm in antibiotic resistance and food contamination. Int J Microbiol 2020:1705814. doi:10.1155/2020/1705814 32908520 PMC7468660

[21] Singh S, Singh SK, Chowdhury I, Singh R. 2017. Understanding the mechanism of bacterial biofilms resistance to antimicrobial agents. Open Microbiol J 11:53–62. doi:10.2174/1874285801711010053 28553416 PMC5427689

[22] Yousra T, Mehri I, Lajnef R, Rejab AB, Khessairi A, Cherif H, Ouzari H, Hassen A. 2017. Biofilms in bioremediation and wastewater treatment: characterization of bacterial community structure and diversity during seasons in municipal wastewater treatment process. Environ Sci Pollut Res 24:3519–3530. doi:10.1007/s11356-016-8090-2 27878485

[23] Saini S, Tewari S, Dwivedi J, Sharma V. 2023. Biofilm-mediated wastewater treatment: a comprehensive review. Mater Adv 4:1415–1443. doi:10.1039/D2MA00945E

[24] Maurya A, Kumar R, Raj A. 2023. Biofilm-based technology for industrial wastewater treatment: current technology, applications and future perspectives. World J Microbiol Biotechnol 39:112. doi:10.1007/s11274-023-03567-7 36907929

[25] Guo X, Li B, Zhao R, Zhang J, Lin L, Zhang G, Li R-H, Liu J, Li P, Li Y, Li X-Y. 2019. Performance and bacterial community of moving bed biofilm reactors with various biocarriers treating primary wastewater effluent with a low organic strength and low C/N ratio. Bioresour Technol 287:121424. doi:10.1016/j.biortech.2019.121424 31082673

[26] Cockell CS, Bush T, Bryce C, Direito S, Fox-Powell M, Harrison JP, Lammer H, Landenmark H, Martin-Torres J, Nicholson N, Noack L, O’Malley-James J, Payler SJ, Rushby A, Samuels T, Schwendner P, Wadsworth J, Zorzano MP. 2016. Habitability: a review. Astrobiology 16:89–117. doi:10.1089/ast.2015.1295 26741054

[27] Williams HN, Turng BF, Kelley JI. 2009. Survival response of bacteriovorax in surface biofilm versus suspension when stressed by extremes in environmental conditions. Microb Ecol 58:474–484. doi:10.1007/s00248-009-9499-7 19267151

[28] Strelkova EA, Pozdnyakova NV, Zhurina MV, Plakunov VK, Belyaev SS. 2013. Role of the extracellular polymer matrix in resistance of bacterial biofilms to extreme environmental factors. Microbiology (Reading, Engl) 82:119–125. doi:10.1134/S002626171302015X 23808136

[29] Knowles EJ, Castenholz RW. 2008. Effect of exogenous extracellular polysaccharides on the desiccation and freezing tolerance of rock-inhabiting phototrophic microorganisms. FEMS Microbiol Ecol 66:261–270. doi:10.1111/j.1574-6941.2008.00568.x 18710394

[30] Weaver L, Webber JB, Hickson AC, Abraham PM, Close ME. 2015. Biofilm resilience to desiccation in groundwater aquifers: a laboratory and field study. Sci Total Environ 514:281–289. doi:10.1016/j.scitotenv.2014.10.031 25668280

[31] Lapaglia C, Hartzell PL. 1997. Stress-induced production of biofilm in the hyperthermophile Archaeoglobus fulgidus. Appl Environ Microbiol 63:3158–3163. doi:10.1128/aem.63.8.3158-3163.1997 16535671 PMC1389226

[32] Merino N, Aronson HS, Bojanova DP, Feyhl-Buska J, Wong ML, Zhang S, Giovannelli D. 2019. Living at the extremes: extremophiles and the limits of life in a planetary context. Front Microbiol 10:780. doi:10.3389/fmicb.2019.00780 31037068 PMC6476344

[33] Roberson EB, Firestone MK. 1992. Relationship between desiccation and exopolysaccharide production in a soil Pseudomonas sp. Appl Environ Microbiol 58:1284–1291. doi:10.1128/aem.58.4.1284-1291.1992 16348695 PMC195588

[34] Decho AW, Gutierrez T. 2017. Microbial extracellular polymeric substances (EPSs) in ocean systems. Front Microbiol 8:922. doi:10.3389/fmicb.2017.00922 28603518 PMC5445292

[35] Charles CJ, Rout SP, Patel KA, Akbar S, Laws AP, Jackson BR, Boxall SA, Humphreys PN. 2017. Floc formation reduces the pH stress experienced by microorganisms living in alkaline environments. Appl Environ Microbiol 83:e02985-16. doi:10.1128/AEM.02985-16 28087527 PMC5335526

[36] Aguilera A, Souza-Egipsy V, Martín-Uriz PS, Amils R. 2008. Extracellular matrix assembly in extreme acidic eukaryotic biofilms and their possible implications in heavy metal adsorption. Aquat Toxicol 88:257–266. doi:10.1016/j.aquatox.2008.04.014 18554732

[37] Mozaheb N, Rasouli P, Kaur M, Van Der Smissen P, Larrouy-Maumus G, Mingeot-Leclercq MP. 2023. A Mildly acidic environment alters Pseudomonas aeruginosa virulence and causes remodeling of the bacterial surface. Microbiol Spectr 11:e0483222. doi:10.1128/spectrum.04832-22 37278652 PMC10433952

[38] Tran P, Lander SM, Prindle A. 2024. Active pH regulation facilitates Bacillus subtilis biofilm development in a minimally buffered environment. MBio 15:e0338723. doi:10.1128/mbio.03387-23 38349175 PMC10936434

[39] Yu S, Wei Q, Zhao T, Guo Y, Ma LZ. 2016. A survival strategy for Pseudomonas aeruginosa that uses exopolysaccharides to sequester and store iron to stimulate Psl-Dependent Biofilm Formation. Appl Environ Microbiol 82:6403–6413. doi:10.1128/AEM.01307-16 27565622 PMC5066357

[40] Sun P, Gao M, Sun R, Wu Y, Dolfing J. 2021. Periphytic biofilms accumulate manganese, intercepting its emigration from paddy soil. J Hazard Mater 411:125172. doi:10.1016/j.jhazmat.2021.125172 33858112

[41] Caruso C, Rizzo C, Mangano S, Poli A, Di Donato P, Nicolaus B, Di Marco G, Michaud L, Lo Giudice A. 2018. Extracellular polymeric substances with metal adsorption capacity produced by Pseudoalteromonas sp. MER144 from Antarctic seawater. Environ Sci Pollut Res 25:4667–4677. doi:10.1007/s11356-017-0851-z 29197057

[42] Nocelli N, Bogino PC, Banchio E, Giordano W. 2016. Roles of extracellular polysaccharides and biofilm formation in heavy metal resistance of Rhizobia. Materials (Basel) 9:418. doi:10.3390/ma9060418 28773540 PMC5456807

[43] Gutierrez T, Shimmield T, Haidon C, Black K, Green DH. 2008. Emulsifying and metal ion binding activity of a glycoprotein exopolymer produced by Pseudoalteromonas sp. strain TG12. Appl Environ Microbiol 74:4867–4876. doi:10.1128/AEM.00316-08 18552188 PMC2519319

[44] Bhaskar PV, Bhosle NB. 2006. Bacterial extracellular polymeric substance (EPS): a carrier of heavy metals in the marine food-chain. Environ Int 32:191–198. doi:10.1016/j.envint.2005.08.010 16256198

[45] Hou J, Veeregowda DH, van de Belt-Gritter B, Busscher HJ, van der Mei HC. 2018. extracellular polymeric matrix production and relaxation under fluid shear and mechanical pressure in Staphylococcus aureus biofilms. Appl Environ Microbiol 84:e01516-17. doi:10.1128/AEM.01516-17 29054874 PMC5734043

[46] Liu Q, Yang R, Sun X, Zhou X, Chen H. 2023. Biofilm formation under high temperature causes the commensal bacteria Bacillus cereus WPySW2 to shift from friend to foe in Neoporphyra haitanensis in vitro model. J Ocean Limnol 41:229–240. doi:10.1007/s00343-022-1339-3

[47] Caruso C, Rizzo C, Mangano S, Poli A, Di Donato P, Finore I, Nicolaus B, Di Marco G, Michaud L, Lo Giudice A. 2018. Production and biotechnological potential of extracellular polymeric substances from sponge-associated antarctic bacteria. Appl Environ Microbiol 84:e01624-17. doi:10.1128/AEM.01624-17 29180360 PMC5795064

[48] Underwood GJC, Fietz S, Papadimitriou S, Thomas DN, Dieckmann GS. 2010. Distribution and composition of dissolved extracellular polymeric substances (EPS) in Antarctic sea ice. Mar Ecol Prog Ser 404:1–19. doi:10.3354/meps08557

[49] Ali P, Fucich D, Shah AA, Hasan F, Chen F. 2021. Cryopreservation of cyanobacteria and eukaryotic microalgae using exopolysaccharide extracted from a glacier bacterium. Microorganisms 9:395. doi:10.3390/microorganisms9020395 33671910 PMC7918967

[50] Mhatre E, Troszok A, Gallegos-Monterrosa R, Lindstädt S, Hölscher T, Kuipers OP, Kovács ÁT. 2016. The impact of manganese on biofilm development of Bacillus subtilis. Microbiology (Reading) 162:1468–1478. doi:10.1099/mic.0.000320 27267987

[51] Kang D, Kirienko NV. 2018. Interdependence between iron acquisition and biofilm formation in Pseudomonas aeruginosa. J Microbiol 56:449–457. doi:10.1007/s12275-018-8114-3 29948830 PMC6221862

[52] Baqué M, Scalzi G, Rabbow E, Rettberg P, Billi D. 2013. Biofilm and planktonic lifestyles differently support the resistance of the desert cyanobacterium Chroococcidiopsis under space and Martian simulations. Orig Life Evol Biosph 43:377–389. doi:10.1007/s11084-013-9341-6 23955666

[53] Guo Q, Zhan Y, Zhang W, Wang J, Yan Y, Wang W, Lin M. 2024. Development and regulation of the extreme biofilm formation of Deinococcus radiodurans R1 under Extreme environmental conditions. IJMS 25:421. doi:10.3390/ijms25010421 PMC10778927 38203592

[54] Elasri MO, Miller RV. 1999. Study of the response of a biofilm bacterial community to UV radiation. Appl Environ Microbiol 65:2025–2031. doi:10.1128/AEM.65.5.2025-2031.1999 10223995 PMC91292

[55] Billi D, Staibano C, Verseux C, Fagliarone C, Mosca C, Baqué M, Rabbow E, Rettberg P. 2019. Dried biofilms of desert strains of Chroococcidiopsis survived prolonged exposure to space and mars-like conditions in low earth orbit . Astrobiology 19:1008–1017. doi:10.1089/ast.2018.1900 30741568

[56] Panitz C, Frösler J, Wingender J, Flemming HC, Rettberg P. 2019. Tolerances of Deinococcus geothermalis biofilms and planktonic cells exposed to space and simulated martian conditions in low earth orbit for almost two years. Astrobiology 19:979–994. doi:10.1089/ast.2018.1913 30925079

[57] Zhang Q, Nguyen D, Tai JB, Xu XJ, Nijjer J, Huang X, Li Y, Yan J. 2022. mechanical resilience of biofilms toward environmental perturbations mediated by extracellular matrix. Adv Funct Materials 32:2110699. doi:10.1002/adfm.202110699

[58] Zhao X, Yang Y, Feng K, Wang X, Liu B, Xie G, Xing D. 2021. Self-regulating microbiome networks ensure functional resilience of biofilms in sand biofilters during manganese load fluctuations. Water Res 188:116473. doi:10.1016/j.watres.2020.116473 33038718

[59] Feng K, Zhang Z, Cai W, Liu W, Xu M, Yin H, Wang A, He Z, Deng Y. 2017. Biodiversity and species competition regulate the resilience of microbial biofilm community. Mol Ecol 26:6170–6182. doi:10.1111/mec.14356 28926148

[60] Dzubakova K, Peter H, Bertuzzo E, Juez C, Franca MJ, Rinaldo A, Battin TJ. 2018. Environmental heterogeneity promotes spatial resilience of phototrophic biofilms in streambeds. Biol Lett 14:20180432. doi:10.1098/rsbl.2018.0432 30305460 PMC6227859

[61] McKew BA, Taylor JD, McGenity TJ, Underwood GJC. 2011. Resistance and resilience of benthic biofilm communities from a temperate saltmarsh to desiccation and rewetting. ISME J 5:30–41. doi:10.1038/ismej.2010.91 20596071 PMC3105671

[62] Ricciardelli A, Casillo A, Vergara A, Balasco N, Corsaro MM, Tutino ML, Parrilli E. 2019. Environmental conditions shape the biofilm of the Antarctic bacterium Pseudoalteromonas haloplanktis TAC125. Microbiol Res 218:66–75. doi:10.1016/j.micres.2018.09.010 30454660

[63] Jiao Y, Cody GD, Harding AK, Wilmes P, Schrenk M, Wheeler KE, Banfield JF, Thelen MP. 2010. Characterization of extracellular polymeric substances from acidophilic microbial biofilms. Appl Environ Microbiol 76:2916–2922. doi:10.1128/AEM.02289-09 20228116 PMC2863431

[64] Nichols CM, Lardière SG, Bowman JP, Nichols PD, A E Gibson J, Guézennec J. 2005. Chemical characterization of exopolysaccharides from Antarctic marine bacteria. Microb Ecol 49:578–589. doi:10.1007/s00248-004-0093-8 16052372

[65] Tielen P, Strathmann M, Jaeger KE, Flemming HC, Wingender J. 2005. Alginate acetylation influences initial surface colonization by mucoid Pseudomonas aeruginosa. Microbiol Res 160:165–176. doi:10.1016/j.micres.2004.11.003 15881834

[66] Körstgens V, Flemming HC, Wingender J, Borchard W. 2001. Influence of calcium ions on the mechanical properties of a model biofilm of mucoid Pseudomonas aeruginosa. Water Sci Technol 43:49–57. 11381972

[67] Noffke N. 2010. Geobiology: microbial mats in sandy deposits Mats in sandy deposits from the Archean era to today. Springer, Berlin, Heidelberg. Available from: https://link.springer.com/10.1007/978-3-642-12772-4

[68] Krembs C, Eicken H, Deming JW. 2011. Exopolymer alteration of physical properties of sea ice and implications for ice habitability and biogeochemistry in a warmer Arctic. Proc Natl Acad Sci USA 108:3653–3658. doi:10.1073/pnas.1100701108 21368216 PMC3048104

[69] Henao LJ, Mazeau K. 2009. Molecular modelling studies of clay–exopolysaccharide complexes: soil aggregation and water retention phenomena. Mater Sci Eng: C 29:2326–2332. doi:10.1016/j.msec.2009.06.001

[70] Los Ríos A, Wierzchos J, Sancho LG, Ascaso C. 2003. Acid microenvironments in microbial biofilms of antarctic endolithic microecosystems. Environ Microbiol 5:231–237. doi:10.1046/j.1462-2920.2003.00417.x 12662170

[71] Liu X, Eusterhues K, Thieme J, Ciobota V, Höschen C, Mueller CW, Küsel K, Kögel-Knabner I, Rösch P, Popp J, Totsche KU. 2013. STXM and NanoSIMS investigations on EPS fractions before and after adsorption to Goethite. Environ Sci Technol 47:3158–3166. doi:10.1021/es3039505 23451805

[72] Costa OYA, Raaijmakers JM, Kuramae EE. 2018. Microbial extracellular polymeric substances: ecological function and impact on soil aggregation. Front Microbiol 9:1636. doi:10.3389/fmicb.2018.01636 30083145 PMC6064872

[73] Chen XD, Zhang CK, Zhou Z, Gong Z, Zhou JJ, Tao JF, Paterson DM, Feng Q. 2017. Stabilizing effects of bacterial biofilms: EPS penetration and redistribution of bed stability down the sediment profile. JGR Biogeosciences 122:3113–3125. doi:10.1002/2017JG004050

[74] Jones CG, Lawton JH, Shachak M. Organisms as ecosystem engineers. In: Samson FB, Knopf FL, editors. Ecosystem management: selected readings. New York, NY: Springer; 1996. p. 130–47.

[75] Dang H, Lovell CR. 2000. Bacterial primary colonization and early succession on surfaces in marine waters as determined by amplified rRNA gene restriction analysis and sequence analysis of 16S rRNA genes. Appl Environ Microbiol 66:467–475. doi:10.1128/AEM.66.2.467-475.2000 10653705 PMC91850

[76] Petrova OE, Sauer K. 2012. Sticky situations: key components that control bacterial surface attachment. J Bacteriol 194:2413–2425. doi:10.1128/JB.00003-12 22389478 PMC3347170

[77] Bowden GHW, Li YH. 1997. Nutritional influences on biofilm development. Adv Dent Res 11:81–99. doi:10.1177/08959374970110012101 9524446

[78] Zhang W, Seminara A, Suaris M, Brenner MP, Weitz DA, Angelini TE. 2014. Nutrient depletion in Bacillus subtilis biofilms triggers matrix production. New J Phys 16:015028. doi:10.1088/1367-2630/16/1/015028

[79] Wang J, Peipoch M, Guo X, Kan J. 2022. Convergence of biofilm successional trajectories initiated during contrasting seasons. Front Microbiol 13:991816. doi:10.3389/fmicb.2022.991816 36187986 PMC9522907

[80] Lee YM, Cho KH, Hwang K, Kim EH, Kim M, Hong SG, Lee HK. 2016. Succession of bacterial community structure during the early stage of biofilm development in the Antarctic marine environment. Korean J Microbiol 52:49–58. doi:10.7845/kjm.2016.6005

[81] Holmström C, Kjelleberg S. 1999. Marine Pseudoalteromonas species are associated with higher organisms and produce biologically active extracellular agents. FEMS Microbiol Ecol 30:285–293. doi:10.1111/j.1574-6941.1999.tb00656.x 10568837

[82] Teal TK, Lies DP, Wold BJ, Newman DK. 2006. Spatiometabolic stratification of Shewanella oneidensis biofilms. Appl Environ Microbiol 72:7324–7330. doi:10.1128/AEM.01163-06 16936048 PMC1636161

[83] Brazelton WJ, Mehta MP, Kelley DS, Baross JA. 2011. Physiological differentiation within a single-species biofilm fueled by serpentinization. MBio 2:00127–11. doi:10.1128/mBio.00127-11 PMC3143844 21791580

[84] Han M, Zhu X, Ruan C, Wu H, Chen G, Zhu K, Liu Y, Wang G. 2024. Micro-biophysical interactions at bacterium-mineral interfaces determine potassium dissolution. Environ Technol & Innov 33:103524. doi:10.1016/j.eti.2023.103524

[85] Guido A, Sposato M, Palladino G, Vescogni A, Miriello D. 2022. Biomineralization of primary carbonate cements: a new biosignature in the fossil record from the Anisian of Southern Italy. LET 55:1–21. doi:10.1111/let.12450

[86] Ehrlich HL. 1996. How microbes influence mineral growth and dissolution. Chem Geol 132:5–9. doi:10.1016/S0009-2541(96)00035-6

[87] Boquet E, Boronat A, Ramos-cormenzana A. 1973. Production of calcite (calcium carbonate) crystals by soil bacteria is a general phenomenon. Nature New Biol 246:527–529. doi:10.1038/246527a0

[88] Wei S, Cui H, Jiang Z, Liu H, He H, Fang N. 2015. Biomineralization processes of calcite induced by bacteria isolated from marine sediments. Braz J Microbiol 46:455–464. doi:10.1590/S1517-838246220140533 26273260 PMC4507537

[89] Martin W, Russell MJ. 2007. On the origin of biochemistry at an alkaline hydrothermal vent. Philos Trans R Soc Lond B Biol Sci 362:1887–1925. doi:10.1098/rstb.2006.1881 17255002 PMC2442388

[90] Aquino KA, Früh‐Green GL, Bernasconi SM, Bontognali TRR, Foubert A, Lang SQ. 2024. Controls on mineral formation in high pH fluids from the lost city hydrothermal field. Geochem Geophys Geosyst 25:e2023GC011010. doi:10.1029/2023GC011010

[91] Pisapia C, Gérard E, Gérard M, Lecourt L, Lang SQ, Pelletier B, Payri CE, Monnin C, Guentas L, Postec A, Quéméneur M, Erauso G, Ménez B. 2017. Mineralizing filamentous bacteria from the Prony Bay hydrothermal field give new insights into the functioning of serpentinization-based subseafloor ecosystems. Front Microbiol 8:57. doi:10.3389/fmicb.2017.00057 28197130 PMC5281578

[92] Lingappa UF, Yeager CM, Sharma A, Lanza NL, Morales DP, Xie G, Atencio AD, Chadwick GL, Monteverde DR, Magyar JS, Webb SM, Valentine JS, Hoffman BM, Fischer WW. 2021. An ecophysiological explanation for manganese enrichment in rock varnish. Proc Natl Acad Sci USA 118:e2025188118. doi:10.1073/pnas.2025188118 34161271 PMC8237629

[93] Yin X, Weitzel F, Griesshaber E, Fernández-Díaz L, Jimenez-Lopez C, Ziegler A, et al.. 2020. Bacterial EPS in agarose hydrogels directs mineral organization in calcite precipitates: species-specific biosignatures of Bacillus subtilis, Mycobacterium phley, Mycobacterium smagmatis, and Pseudomonas putida EPS. Cryst Growth Des 20:4402–4417. doi:10.1021/acs.cgd.0c00231

[94] Culotta VC, Wildeman AS. 2021. Shining light on photosynthetic microbes and manganese-enriched rock varnish. Proc Natl Acad Sci USA 118:e2109436118. doi:10.1073/pnas.2109436118 34183441 PMC8285942

[95] Lanza NL, Wiens RC, Arvidson RE, Clark BC, Fischer WW, Gellert R, Grotzinger JP, Hurowitz JA, McLennan SM, Morris RV, et al.. 2016. Oxidation of manganese in an ancient aquifer, Kimberley formation, Gale crater, Mars. Geophys Res Lett 43:7398–7407. doi:10.1002/2016GL069109

[96] Perry RS, Kolb VM. 2004. Biological and organic constiuents of desert varnish: review and new hypotheses. Instruments, methods, and missions for astrobiology VII:202–217. doi:10.1117/12.509695

[97] Dupraz C, Visscher PT, Baumgartner LK, Reid RP. 2004. Microbe–mineral interactions: early carbonate precipitation in a hypersaline lake (Eleuthera Island, Bahamas). Sedimentology 51:745–765. doi:10.1111/j.1365-3091.2004.00649.x

[98] Hall-Stoodley L, Costerton JW, Stoodley P. 2004. Bacterial biofilms: from the natural environment to infectious diseases. Nat Rev Microbiol 2:95–108. doi:10.1038/nrmicro821 15040259

[99] Egel R. 2014. Origins and emergent evolution of life: the colloid microsphere hypothesis revisited. Orig Life Evol Biosph 44:87–110. doi:10.1007/s11084-014-9363-8 25208738

[100] Cairns-Smith AG. 1966. The origin of life and the nature of the primitive gene. J Theor Biol 10:53–88. doi:10.1016/0022-5193(66)90178-0 5964688

[101] Trevors JT. 2011. Hypothesized origin of microbial life in a prebiotic gel and the transition to a living biofilm and microbial mats. C R Biol 334:269–272. doi:10.1016/j.crvi.2011.02.010 21513895

[102] Römling U. 2023. Is biofilm formation intrinsic to the origin of life? Environ Microbiol 25:26–39. doi:10.1111/1462-2920.16179 36655713 PMC10086821

[103] Baross JA, Hoffman SE. 1985. Submarine hydrothermal vents and associated gradient environments as sites for the origin and evolution of life. Origins Life Evol Biosphere 15:327–345. doi:10.1007/BF01808177

[104] Woese C. 1998. The universal ancestor. Proc Natl Acad Sci USA 95:6854–6859. doi:10.1073/pnas.95.12.6854 9618502 PMC22660

[105] Trevors JT, Pollack GH. 2005. Hypothesis: the origin of life in a hydrogel environment. Prog Biophys Mol Biol 89:1–8. doi:10.1016/j.pbiomolbio.2004.07.003 15826671

[106] Szostak JW. 2016. On the origin of life. Medicina (B Aires) 76:199–203. 27576276

[107] Dalai P, Sahai N. 2019. Mineral–lipid interactions in the origins of life. Trends Biochem Sci 44:331–341. doi:10.1016/j.tibs.2018.11.009 30583961

[108] Hazen RM, Sverjensky DA. 2010. Mineral surfaces, geochemical complexities, and the origins of life. Cold Spring Harb Perspect Biol 2:a002162. doi:10.1101/cshperspect.a002162 20452963 PMC2857174

[109] Churchill H, Teng H, Hazen RM. 2004. Correlation of pH-dependent surface interaction forces to amino acid adsorption: implications for the origin of life. Am Mineral 89:1048–1055. doi:10.2138/am-2004-0716

[110] Dong H, Huang L, Zhao L, Zeng Q, Liu X, Sheng Y, Shi L, Wu G, Jiang H, Li F, Zhang L, Guo D, Li G, Hou W, Chen H. 2022. A critical review of mineral-microbe interaction and co-evolution: mechanisms and applications. Natl Sci Rev 9:nwac128. doi:10.1093/nsr/nwac128 36196117 PMC9522408

[111] Zeng G, Vad BS, Dueholm MS, Christiansen G, Nilsson M, Tolker-Nielsen T, Nielsen PH, Meyer RL, Otzen DE. 2015. Functional bacterial amyloid increases Pseudomonas biofilm hydrophobicity and stiffness. Front Microbiol 6:1099. doi:10.3389/fmicb.2015.01099 26500638 PMC4595789

[112] Fändrich M. 2007. On the structural definition of amyloid fibrils and other polypeptide aggregates. Cell Mol Life Sci 64:2066–2078. doi:10.1007/s00018-007-7110-2 17530168 PMC11138455

[113] Romero D, Aguilar C, Losick R, Kolter R. 2010. Amyloid fibers provide structural integrity to Bacillus subtilis biofilms . Proc Natl Acad Sci USA 107:2230–2234. doi:10.1073/pnas.0910560107 20080671 PMC2836674

[114] Des Marais DJ, Nuth JA 3rd, Allamandola LJ, Boss AP, Farmer JD, Hoehler TM, Jakosky BM, Meadows VS, Pohorille A, Runnegar B, Spormann AM. 2008. The NASA Astrobiology Roadmap. Astrobiology 8:715–730. doi:10.1089/ast.2008.0819 18793098

[115] Glamoclija M, Garrel L, Berthon J, López-García P. 2004. Biosignatures and bacterial diversity in hydrothermal deposits of solfatara crater, Italy. Geomicrobiol J 21:529–541. doi:10.1080/01490450490888235

[116] del Buey P, Sanz-Montero ME, Braissant O, Cabestrero Ó, Visscher PT. 2021. The role of microbial extracellular polymeric substances on formation of sulfate minerals and fibrous Mg-clays. Chem Geol 581:120403. doi:10.1016/j.chemgeo.2021.120403

[117] Bosak T, Moore KR, Gong J, Grotzinger JP. 2021. Searching for biosignatures in sedimentary rocks from early earth and mars. Nat Rev Earth Environ 2:490–506. doi:10.1038/s43017-021-00169-5

[118] Gorbushina AA, Krumbein WE, Volkmann M. 2002. Rock surfaces as life indicators: new ways to demonstrate life and traces of former life. Astrobiology 2:203–213. doi:10.1089/15311070260192273 12469369

[119] Cámara B, Souza-Egipsy V, Ascaso C, Artieda O, De Los Ríos A, Wierzchos J. 2016. Biosignatures and microbial fossils in endolithic microbial communities colonizing Ca-sulfate crusts in the Atacama desert. Chem Geol 443:22–31. doi:10.1016/j.chemgeo.2016.09.019

[120] Banfield JF, Moreau JW, Chan CS, Welch SA, Little B. 2001. Mineralogical biosignatures and the search for life on Mars. Astrobiology 1:447–465. doi:10.1089/153110701753593856 12448978

[121] Kleber M, Eusterhues K, Keiluweit M, Mikutta C, Mikutta R, Nico PS. 2015. Chapter one - mineral–organic associations: formation, properties, and relevance in soil environments, p 1–140. In Sparks DL (ed), Advances in agronomy. Academic Press.

[122] Duteil T, Bourillot R, Braissant O, Grégoire B, Leloup M, Portier E, Brigaud B, Féniès H, Svahn I, Henry A, Yokoyama Y, Visscher PT. 2022. Preservation of exopolymeric substances in estuarine sediments. Front Microbiol 13:921154. doi:10.3389/fmicb.2022.921154 36060749 PMC9434125

[123] Schopf JW. 1993. Microfossils of the early archean apex chert: new evidence of the antiquity of life. Science 260:640–646. doi:10.1126/science.260.5108.640 11539831

[124] Westall F, de Wit MJ, Dann J, van der Gaast S, de Ronde CEJ, Gerneke D. 2001. Early Archean fossil bacteria and biofilms in hydrothermally-influenced sediments from the Barberton greenstone belt, South Africa. Precambrian Res 106:93–116. doi:10.1016/S0301-9268(00)00127-3

[125] Rasmussen B. 2000. Filamentous microfossils in a 3,235-million-year-old volcanogenic massive sulphide deposit. Nature 405:676–679. doi:10.1038/35015063 10864322

[126] Casar CP, Kruger BR, Osburn MR. 2021. Rock-hosted subsurface biofilms: mineral selectivity drives hotspots for intraterrestrial life. Front Microbiol 12:658988. doi:10.3389/fmicb.2021.658988 33897673 PMC8062869

[127] Jones AA, Bennett PC. 2014. Mineral microniches control the diversity of subsurface microbial populations. Geomicrobiol J 31:246–261. doi:10.1080/01490451.2013.809174

[128] Briggs BR, Pohlman JW, Torres M, Riedel M, Brodie EL, Colwell FS. 2011. Macroscopic biofilms in fracture-dominated sediment that anaerobically oxidize methane. Appl Environ Microbiol 77:6780–6787. doi:10.1128/AEM.00288-11 21821755 PMC3187087

[129] Templeton AS, Ellison ET, Glombitza C, Morono Y, Rempfert KR, Hoehler TM, Zeigler SD, Kraus EA, Spear JR, Nothaft DB, Fones EM, Boyd ES, Munro‐Ehrlich M, Mayhew LE, Cardace D, Matter JM, Kelemen PB, the Oman Drilling Project Science Party. 2021. Accessing the subsurface biosphere within rocks undergoing active low‐temperature serpentinization in the samail ophiolite (Oman Drilling Project). JGR Biogeosciences 126. doi:10.1029/2021JG006315

[130] Hickman AH. 2012. Review of the pilbara craton and fortescue basin, Western Australia: crustal evolution providing environments for early life. Island Arc 21:1–31. doi:10.1111/j.1440-1738.2011.00783.x

[131] Noffke N, Christian D, Wacey D, Hazen RM. 2013. Microbially induced sedimentary structures recording an ancient ecosystem in the ca. 3.48 billion-year-old dresser formation, Pilbara, Western Australia. Astrobiology 13:1103–1124. doi:10.1089/ast.2013.1030 24205812 PMC3870916

[132] Noffke N. 2021. Microbially Induced sedimentary structures in clastic deposits: implication for the prospection for fossil life on Mars. Astrobiology 21:866–892. doi:10.1089/ast.2021.0011 34042490 PMC8262410

[133] Hickman-Lewis K, Cavalazzi B, Foucher F, Westall F. 2018. Most ancient evidence for life in the Barberton greenstone belt: microbial mats and biofabrics of the ∼3.47 Ga Middle Marker horizon. Precambrian Res 312:45–67. doi:10.1016/j.precamres.2018.04.007

[134] Homann M. 2019. Earliest life on earth: evidence from the barberton greenstone belt, South Africa. Earth Sci Rev 196:102888. doi:10.1016/j.earscirev.2019.102888

[135] Aguilera A, Souza-Egipsy V, Gómez F, Amils R. 2007. Development and structure of eukaryotic biofilms in an extreme acidic environment, rio tinto (SW, Spain). Microb Ecol 53:294–305. doi:10.1007/s00248-006-9092-2 17268880

[136] Amils R, González-Toril E, Fernández-Remolar D, Gómez F, Aguilera Á, Rodríguez N, Malki M, García-Moyano A, Fairén AG, de la Fuente V, Luis Sanz J. 2007. Extreme environments as Mars terrestrial analogs: the rio tinto case. Planet Space Sci 55:370–381. doi:10.1016/j.pss.2006.02.006

[137] Azua-Bustos A, González-Silva C, Fairén AG. 2022. The Atacama desert in northern Chile as an analog model of mars. Front Astron Space Sci 8. doi:10.3389/fspas.2021.810426

[138] Vignale FA, Kurth D, Lencina AI, Poiré DG, Chihuailaf E, Muñoz-Herrera NC, Novoa F, Contreras M, Turjanski AG, Farías ME. 2021. Geobiology of andean microbial ecosystems discovered in Salar de Atacama, Chile. Front Microbiol 12:762076. doi:10.3389/fmicb.2021.762076 34777316 PMC8581658

[139] Azúa-Bustos A, González-Silva C, Mancilla RA, Salas L, Palma RE, Wynne JJ, McKay CP, Vicuña R. 2009. Ancient photosynthetic eukaryote biofilms in an Atacama desert coastal cave. Microb Ecol 58:485–496. doi:10.1007/s00248-009-9500-5 19259626

[140] Cassaro A, Pacelli C, Aureli L, Catanzaro I, Leo P, Onofri S. 2021. Antarctica as a reservoir of planetary analogue environments. Extremophiles 25:437–458. doi:10.1007/s00792-021-01245-w 34586500

[141] De Los RÃ­os A, Grube M, Sancho LG, Ascaso C. 2007. Ultrastructural and genetic characteristics of endolithic cyanobacterial biofilms colonizing Antarctic granite rocks. FEMS Microbiol Ecol 59:386–395. doi:10.1111/j.1574-6941.2006.00256.x 17328119

[142] Lang SQ, Brazelton WJ. 2020. Habitability of the marine serpentinite subsurface: a case study of the Lost City hydrothermal field. Phil Trans R Soc A 378:20180429. doi:10.1098/rsta.2018.0429 31902336 PMC7015304

[143] Schrenk MO, Kelley DS, Bolton SA, Baross JA. 2004. Low archaeal diversity linked to subseafloor geochemical processes at the lost city hydrothermal field, mid-atlantic ridge. Environ Microbiol 6:1086–1095. doi:10.1111/j.1462-2920.2004.00650.x 15344934

[144] Coelho LF, Blais M-A, Matveev A, Keller-Costa T, Vincent WF, Costa R, Martins Z, Canário J. 2022. Contamination analysis of Arctic ice samples as planetary field analogs and implications for future life-detection missions to Europa and Enceladus. Sci Rep 12:12379. doi:10.1038/s41598-022-16370-5 35896693 PMC9329357

[145] Mohit V, Culley A, Lovejoy C, Bouchard F, Vincent WF. 2017. Hidden biofilms in a far northern lake and implications for the changing Arctic. NPJ Biofilms Microbiomes 3:17. doi:10.1038/s41522-017-0024-3 28702216 PMC5500582

[146] Wagner M, Ivleva NP, Haisch C, Niessner R, Horn H. 2009. Combined use of confocal laser scanning microscopy (CLSM) and Raman microscopy (RM): investigations on EPS-Matrix. Water Res 43:63–76. doi:10.1016/j.watres.2008.10.034 19019406

[147] Wilson C, Lukowicz R, Merchant S, Valquier-Flynn H, Caballero J, Sandoval J, Okuom M, Huber C, Brooks TD, Wilson E, Clement B, Wentworth CD, Holmes AE. 2017. Quantitative and qualitative assessment methods for biofilm growth: a mini-review. Res Rev J Eng Technol 6:http://www.rroij.com/open-access/quantitative-and-qualitative-assessment-methods-for-biofilm-growth-a-minireview-.pdf. PMC6133255 30214915

[148] Edmunson J, Gaskin JA, Jerman GA, Harvey RP, Doloboff IJ, Neidholdt EL. 2016. A miniaturized variable pressure scanning electron microscope (MVP-SEM) for In-Situ mars surface sample analysis. The Woodlands, TX.

[149] Smith HJ, Schmit A, Foster R, Littman S, Kuypers MM, Foreman CM. 2016. Biofilms on glacial surfaces: hotspots for biological activity. NPJ Biofilms Microbiomes 2:16008. doi:10.1038/npjbiofilms.2016.8 28721245 PMC5515272

[150] Sánchez-García L, Carrizo D, Molina A, Muñoz-Iglesias V, Lezcano MÁ, Fernández-Sampedro M, Parro V, Prieto-Ballesteros O. 2020. Fingerprinting molecular and isotopic biosignatures on different hydrothermal scenarios of Iceland, an acidic and sulfur-rich Mars analog. Sci Rep 10:21196. doi:10.1038/s41598-020-78240-2 33273669 PMC7712778

[151] Mountcastle SE, Vyas N, Villapun VM, Cox SC, Jabbari S, Sammons RL, Shelton RM, Walmsley AD, Kuehne SA. 2021. Biofilm viability checker: An open-source tool for automated biofilm viability analysis from confocal microscopy images. NPJ Biofilms Microbiomes 7:44. doi:10.1038/s41522-021-00214-7 33990612 PMC8121819

[152] Flores P, Luo J, Mueller DW, Muecklich F, Zea L. 2024. Space biofilms – An overview of the morphology of Pseudomonas aeruginosa biofilms grown on silicone and cellulose membranes on board the international space station. Biofilm 7:100182. doi:10.1016/j.bioflm.2024.100182 38370151 PMC10869243

[153] Ramirez-Mora T, Dávila-Pérez C, Torres-Méndez F, Valle-Bourrouet G. 2019. Raman Spectroscopic characterization of endodontic biofilm matrices. J Spectrosc (Hindawi) 2019:1–7. doi:10.1155/2019/1307397

[154] Paulo C, Dittrich M. 2013. 2D Raman spectroscopy study of dolomite and cyanobacterial extracellular polymeric substances from Khor Al‐Adaid sabkha (Qatar). J Raman Spectroscopy 44:1563–1569. doi:10.1002/jrs.4368

[155] Bhartia R, Beegle LW, DeFlores L, Abbey W, Razzell Hollis J, Uckert K, Monacelli B, Edgett KS, Kennedy MR, Sylvia M, et al.. 2021. Perseverance’s scanning habitable environments with raman and luminescence for organics and chemicals (SHERLOC) investigation. Space Sci Rev 217:58. doi:10.1007/s11214-021-00812-z

[156] Osterhout JT, Schopf JW, Kudryavtsev AB, Czaja AD, Williford KH. 2022. Deep-UV Raman spectroscopy of Carbonaceous precambrian microfossils: insights into the search for past life on Mars. Astrobiology 22:1239–1254. doi:10.1089/ast.2021.0135 36194869

[157] Sharma G, Prakash A. 2014. Combined use of fourier transform infrared and Raman spectroscopy to study planktonic and biofilm cells of Cronobacter sakazakii. J Microbiol Biotechnol Food sci 3:310–314.

[158] Gordon PR, Sephton MA. 2016. Organic matter detection on mars by pyrolysis-ftir: an analysis of sensitivity and mineral matrix effects. Astrobiology 16:831–845. doi:10.1089/ast.2016.1485 27870586 PMC5124741

[159] Preston LJ, Melim LA, Polyak VJ, Asmerom Y, Southam G. 2014. Infrared spectroscopic biosignatures from hidden cave, new mexico: possible applications for remote life detection. Geomicrobiol J 31:929–941. doi:10.1080/01490451.2014.913096

